# Full-space trifunctional metasurface with independent control of amplitude and phase for circularly polarized waves

**DOI:** 10.1515/nanoph-2024-0441

**Published:** 2024-10-23

**Authors:** Xi Ming Li, Yuan Zhao, Ren Pan Lu, Xiao Feng Sun, Zhao Yang, Hai Dan He, Yan Hui Liu, Guo Hong Du

**Affiliations:** School of Electronic Science and Engineering, University of Electronic Science and Technology of China, Chengdu 611731, China; College of Electronic Engineering, Chengdu University of Information Technology, Chengdu 610225, China; Southwest China Institute of Electronic Technology, Chengdu 610036, China

**Keywords:** multifunctional metasurfaces, amplitude and phase control, full space, wavefront manipulations

## Abstract

Flexible and diverse manipulation of electromagnetic (EM) waves in half space (reflection or transmission) has facilitated strong aspiration toward full-space wave control. However, it remains challenging to achieve independent amplitude and phase control, which seriously hinder the real-world applications. Herein, an innovative strategy of trifunctional metasurface is proposed to independently and simultaneously manipulate the amplitude and phase of circular polarized waves in full space. The multifunctional design is composed of double-layer anisotropic metasurface sandwiched with a bandpass frequency selective surface, with a frequency-direction multiplexed paradigm for on-demand control of both amplitude and phase across three independent channels. To validate the concept, a multifunctional metadevice is designed and verified by simulations and experiments, showcasing arbitrary near-field and far-field power modulation in full space. Lateral and axial bifocal metalenses with desired intensity distribution are designed in two reflection channels at 9 GHz, while multibeam generator with desired spatial scatterings and power allocations is designed in transmissive channel at 13 GHz. The finding paves the way for attaining multifunctional metadevices with amplitude and phase modulation in full space, which have potential applications in high-quality imaging and high-capacity communication systems.

## 1 Introduction

The emergence of metamaterials established a new era for the scientific community, which can produce many exciting phenomena and novel devices that do not exist in nature, such as negative refraction [1], perfect lens [2], and invisibility cloak [3], [4]. Metasurfaces are planar versions of metamaterials, eliminate the bulk of metamaterials, and have shown unprecedented abilities to manipulate the electromagnetic (EM) waves. A plethora of novel phenomena and applications have been discovered by using metasurfaces, such as perfect absorber [5], ultrathin metalens [6], [7], ultrathin cloak [8], [9], vortex beam generator [10], super-reflector [11], low-sidelobe antenna [12], and many others [13], [14].

With the rapid development of modern integration systems, multifunctional devices are required in many applications [15], [16]. Reconfigurable and active metasurfaces can achieve multiple functionalities by incorporating with tunable components such as PIN diodes [17], varactor diodes [18], graphene [19], micro-electromechanical systems [20], or with mechanically tunable structures [21], [22]. The operating frequency can range from the microwave domain [23] to the optical region [24]. However, active metasurfaces usually require complicated designs and an additional control system [25], [26]. It is still a challenge to achieve multiple functionalities with a single passive metasurface. A common strategy is to use anisotropic meta-atoms, which exhibit polarization, helicity, frequency, and propagation direction-controlled phase responses, yielding numerous multifunctional metadevices [15], [27], [28], [29], [30]. The multifunctional metadevices either in reflection (R) [31], or transmission (T) [32], or even full space [33], [34], [35], [36], geometry under either linearly polarized (LP) [37], or circularly polarized (CP) [38] waves excitation.

Despite the fruitful achievements, most aforementioned metasurfaces are based on phase-only modulation. It is well known that amplitude is one of the important characteristics of EM waves, which can be as an additional degree of freedom to EM waves modulation. In many practical applications, however, the EM responses are required under full manipulation for both amplitude and phase (A–P), such as manipulation of multiple diffractions beams [39], high-quality holography generation [40], and synthesis of complex beams [41]. This poses a challenge for conventional metasurface design since the A–P are usually coupled. To circumvent this limitation, some efforts have been explored to implement independent A–P control [42], [43]. For example, split ring resonance structures are proposed to control A–P response of cross-LP wavefronts by adjusting the opening and orientation angles [44], [45], [46]. Such a concept can be further extended from R to T or full-space geometry [47], [48]. However, most of the reported metasurfaces with A–P manipulation are conducted based on LP wave, which leaves complete A–P control of CP wave unexploited. The CP wave is an exclusive polarization state that is extremely crucial for EM devices. Thus, the latest efforts started to be in the area of CP wave with A–P manipulation, such as dual geometric phases [49], hybrid phase [50], [51], and lossy components [52], [53]. Nevertheless, the scattering field of majority of metasurfaces exists in either the R or T half space. Even though the full-space metasurfaces with A–P modulation have recently been investigated under the CP incidence, they are still with the issues of complicated design, high profile, and low efficiency [54], [55], [56].

**Figure 1:**
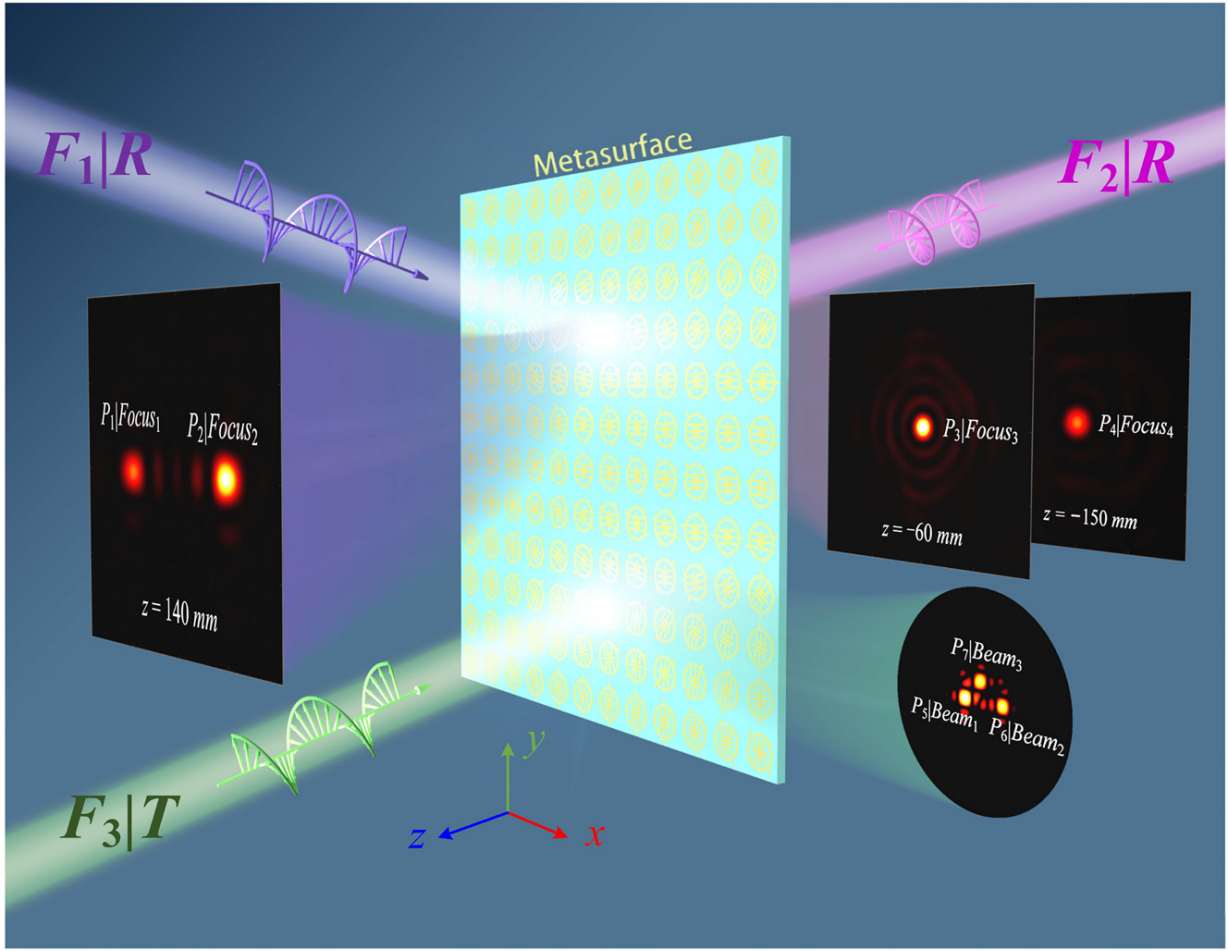
Schematic of the trifunctional metasurface with A–P control in full space. In the reflection mode at *f*_1_, the metasurface behaves as a lateral (or axial) bifocal metalens with desired energy distribution under forward (or backward) RCP wave excitation (functions *F*_1_ and *F*_2_). In the transmission mode at *f*_2_, the metasurface behaves as a multibeam generator with desired spatial scatterings and power allocations under forward RCP wave excitation (function *F*_3_).

In this work, we propose a novel trifunctional metasurface to achieve independent A–P control of CP wave in full space. The proposed meta-atom consists of three different metallic layers printed on both sides of two substrates. The top and bottom metallic layers are composed of two different subelements, which enable external subelements control R channels on both sides of the metasurface at *f*_1_ and internal subelement control T channel at *f*_2_. Besides, a hybrid phase approach synthesizing propagation and geometric phases is employed to achieve independent and arbitrary control of A–P response in R channel, while the modulation of arbitrary A–P response in T channel is accomplished by a dual geometric phases approach. As a demonstration, we experimentally realize a trifunctional metasurface that exhibits three advanced functionalities, and the conceptual illustration is shown in Figure 1. The metasurface can realize desired lateral and axial focal spots with different powers (denoted by *P*_1_–*P*_4_) in R channels, whereas free control scattering directions and power levels (denoted by *P*_5_–*P*_7_) of multiple beams in T channel. Our work paves the way for multifunctional metadevices with A–P modulations in full space, which may flourish the research on metasurfaces.

## 2 Theoretical analysis and meta-atom design

### 2.1 Principle of the independent amplitude and phase modulation

The principle behind our proposed full-space metasurface with independent A–P control is to impose different A–P modulation schemes into three CP channels. Here, a hybrid phase (propagation and geometric phases) strategy is employed to achieve independent A–P control of reflected CP wave, while dual geometric phases approach is employed for A–P control of transmitted CP wave. We discuss principle starting from geometric phase. For any polarized plane wave incident on a R or T meta-atom, the corresponding R or T matrix in LP basis can be expressed as

(1) $$R_{\text{lin}}=\left[\begin{array}{cc} r_{xx} & r_{xy}\\ r_{yx} & r_{yy} \end{array}\right],\;T_{\text{lin}}=\left[\begin{array}{cc} t_{xx} & t_{xy}\\ t_{yx} & t_{yy} \end{array}\right]$$

where *x* and *y* denote the two principal axes, the first and second subscripts denote the output and input polarization. For a meta-atom with mirror symmetry, the scattering coefficient of the meta-atom with a rotation angle *α* under excitation of CP wave can be calculated as (Section S1, Supporting Information)

(2a) $$\begin{array}{ll} R_{\text{cir}}\left(\alpha \right) & =\left[\begin{array}{cc} r_{LL}\left(\alpha \right) & r_{LR}\left(\alpha \right)\\ r_{RL}\left(\alpha \right) & r_{RR}\left(\alpha \right) \end{array}\right]\\ & =\frac{1}{2}\left[\begin{array}{cc} \left(r_{xx}-r_{yy}\right)e^{-j2\alpha } & r_{xx}+r_{yy}\\ r_{xx}+r_{yy} & \left(r_{xx}-r_{yy}\right)e^{j2\alpha } \end{array}\right] \end{array}$$

(2b) $$\begin{array}{ll} T_{\text{cir}}\left(\alpha \right) & =\left[\begin{array}{cc} t_{LL}\left(\alpha \right) & t_{LR}\left(\alpha \right)\\ t_{RL}\left(\alpha \right) & t_{RR}\left(\alpha \right) \end{array}\right]\\ & =\frac{1}{2}\left[\begin{array}{cc} t_{xx}+t_{yy} & \left(t_{xx}-t_{yy}\right)e^{j2\alpha }\\ \left(t_{xx}-t_{yy}\right)e^{-j2\alpha } & t_{xx}+t_{yy} \end{array}\right]\; \end{array}$$

Here, we consider a right-hand circular polarization (RCP) wave illuminating a R meta-atom with the condition of $\left|r_{xx}\right|=\left|r_{yy}\right|=1$ . Then the A–P response of the copolarized reflection wave can be derived from Equation (2a) and written as (detailed analysis is given in Section S2, Supporting Information):

(3a) $$\left|r_{RR}\left(\alpha \right)\right|=\left|\sin\left(\Delta \phi /2\right)\right|$$

(3b) $$\varphi_{RR}^{r}\left(\alpha \right)=\left(\Delta \phi +\pi \right)/2+\varphi_{yy}^{r}+2\alpha$$

where $\Delta \phi =\varphi_{xx}^{r}-\varphi_{yy}^{r}$ is the phase difference between *x*- and *y*-polarization states. Moreover, the phase difference Δ*ϕ* is generated by tailoring the structure parameters, which is viewed as propagation phase. We see that copolarized amplitude is tailored by propagation phase. At the same time, the phase distribution of the copolarized wave depends on both propagation and geometric phases. For simplicity, $\varphi_{yy}^{r}$ is fixed to 0°, and the Δ*ϕ* is determined by $\varphi_{xx}^{r}$ . First, the propagation phase Δ*ϕ* is tuned for amplitude control. Then, the geometric phase ( $\varphi_{RR}^{r}=2\alpha$ ) is tailored to counteract the phase change induced by Δ*ϕ* and obtain the desired phase responses.

Supposing a T meta-atom with dual-metal layers, and the rotation angles of the two metal layers are *β*_1_ and *β*_2_, respectively. Then, the total transmissive matrix of the composite meta-atom can be derived as follows, regardless of the coupling between them.

(4) $$T_{\text{cir}}^{\mathrm{total}}=T_{\text{cir}}\left(\beta_{1}\right)\cdot T_{d}\cdot T_{\text{cir}}\left(\beta_{2}\right)$$

where $T_{\text{cir}}\left(\beta_{1}\right)$ and $T_{\text{cir}}\left(\beta_{2}\right)$ are the transmission coefficients of each metal layer, respectively, which can be extracted from Equation (2b). The transmission coefficient of an ideal isotropic dielectric substrate is $T_{d}=\left[1,0;0,1\right]$ . Suppose the incident wave is polarized as RCP, then we immediately achieve cross-polarized coefficient $T_{LR}^{\text{total}}$ of the compound meta-atom as (detailed analysis is given in Section S3, Supporting Information)

(5) $$T_{LR}^{\text{total}}=\frac{1}{4}\left(T_{xx}^{\text{total}}-T_{yy}^{\text{total}}\right)\left(e^{j2\beta_{1}}+e^{j2\beta_{2}}\right)$$

To engineer maximum cross-CP conversion efficiency, we require anisotropic conditions $\left|T_{xx}^{\text{total}}\right|=\left|T_{yy}^{\text{total}}\right|=1$ and $\varphi_{xx}^{\text{total}}-\varphi_{yy}^{\text{total}}=\pm \pi$ . In this case, the A–P response of the cross-CP component $t_{LR}^{\text{total}}$ can be individually engineered as

(6a) $$\left|t_{LR}^{\text{total}}\right|=\cos\left(\Delta \beta \right)$$

(6b) $$\left|\varphi_{LR}^{\text{total}}\right|=\Delta \beta +2\beta_{1}\;$$

where the angle difference Δ*β* = *β*_2_ − *β*_1_. The result shows the A–P of cross-polarized wave are determined by Δ*β* and *β*_1_.

### 2.2 Frequency-direction multiplexed meta-atom design

**Figure 2:**
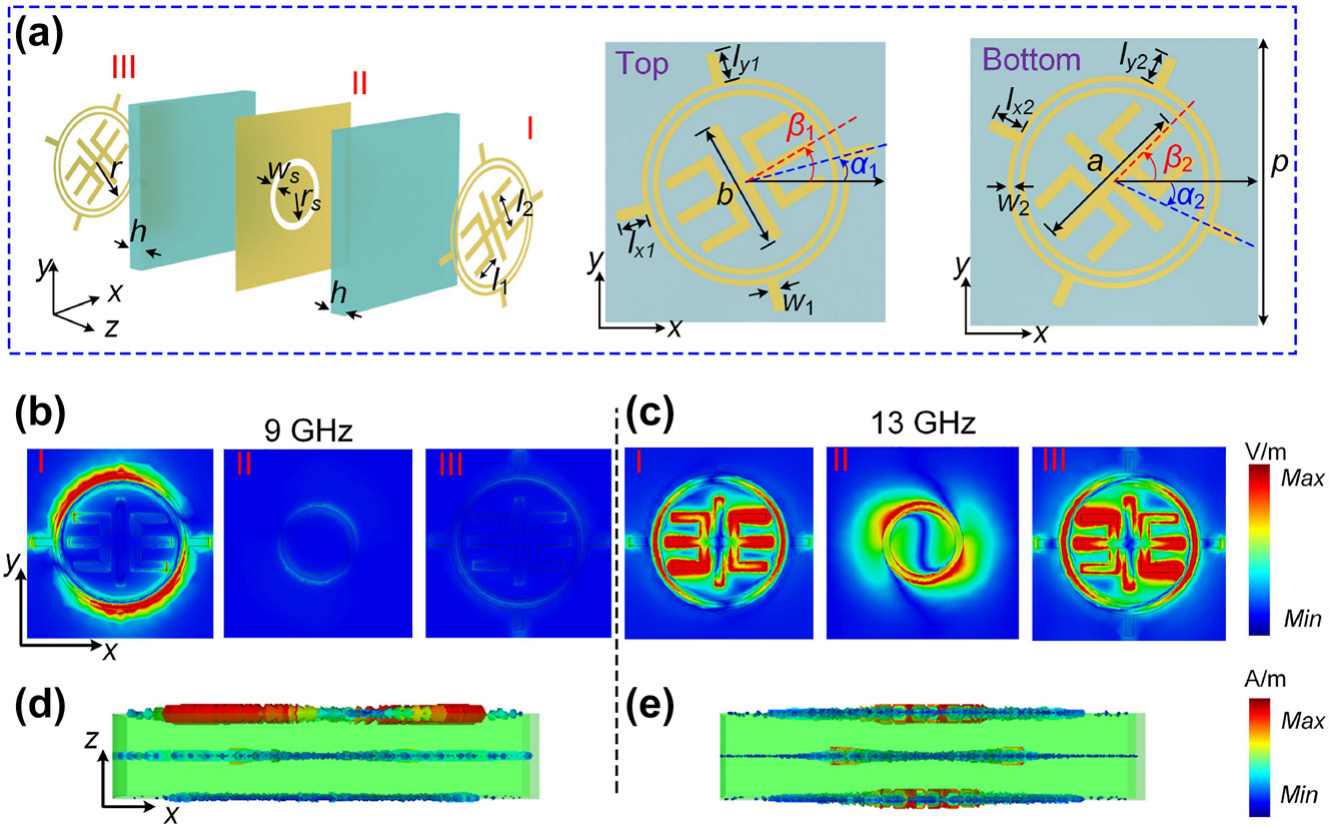
Geometry of the full-space meta-atom with A–P modulation and its EM characteristics. (a) Schematic of the proposed meta-atom. Some geometrical parameters are fixed as *p* = 10, *a* = 5.5, *b* = 4.6, *r* = 3.1, *r*_*s*_ = 2.1, *w*_*s*_ = *w*_2_ = 0.4, *w*_1_ = 0.2, *l*_1_ = 1.88, *l*_2_ = 3, all in the unit of millimeters. (b)–(c) Electric field distribution and (d)–(e) current distribution corresponding to the meta-atom at *f*_1_ and *f*_2_, respectively, under forward RCP wave excitation.

Based on the abovementioned analysis, we design a frequency-direction multiplexed meta-atom for full-space A–P modulation, as shown in Figure 2(a). It comprises three metal layers (I, II, and III) spaced by two F4B substrates (*ɛ*_*r*_ = 2.65, tan* δ* = 0.001) with thickness *h* = 1 mm, exhibiting the ultra-thin property (2 × *h* = 2 mm ≈ 0.06*λ*_0_ and 0.09*λ*_0_ at 9 and 13 GHz, respectively). Two types of concentric subelements are adopted to construct the composite pattern in top and bottom metal layers, say external pattern (the crosshair shaped structure) and internal pattern (symmetrical trident shaped structure). The top and bottom external subelements operate under R mode on both sides of the metasurface, and internal subelement under T mode. To guarantee high insulation among R and T channels, a bandpass frequency selective surface metallic plate etched by circular slot is utilized in the middle layer. It functions as a ground to afford a high reflectivity in R channel at *f*_1_ = 9 GHz, while as a transmissive window for T channel at *f*_2_ = 13 GHz.

To demonstrate the feasibility of our strategy, Figure 2(b) and (c) illustrate the electric field distributions of the meta-atom at different frequencies, under forward RCP wave excitation. In order to guarantee high efficiency at each frequency, we consider the meta-atom with parameters are *l*_*x*1_ = 1.1 mm, *l*_*y*1_ = 0.15 mm, and all rotation angles are zero. The simulations were done in microwave band using commercial software CST Microwave Studio, and we took a unit cell and set periodic boundary conditions in *x*- and *y*-directions. As expected, the field is concentrated on the top external pattern, while there is almost zero intensity on other metal layers at *f*_1_. In sharp contrast, the field mainly concentrates on internal patterns, whereas the external patterns barely responded at *f*_2_. Besides, the current distributions indicate that the CP incident wave can be reflected completely at *f*_1_, while it will pass through the meta-atom at *f*_2_ (Figure 2(d) and (e)), revealing good isolation of dual modes in frequency domain. Because the anisotropic meta-atom can work in both R and T modes, we can realize independent three functions in full space, simultaneously.

**Figure 3:**
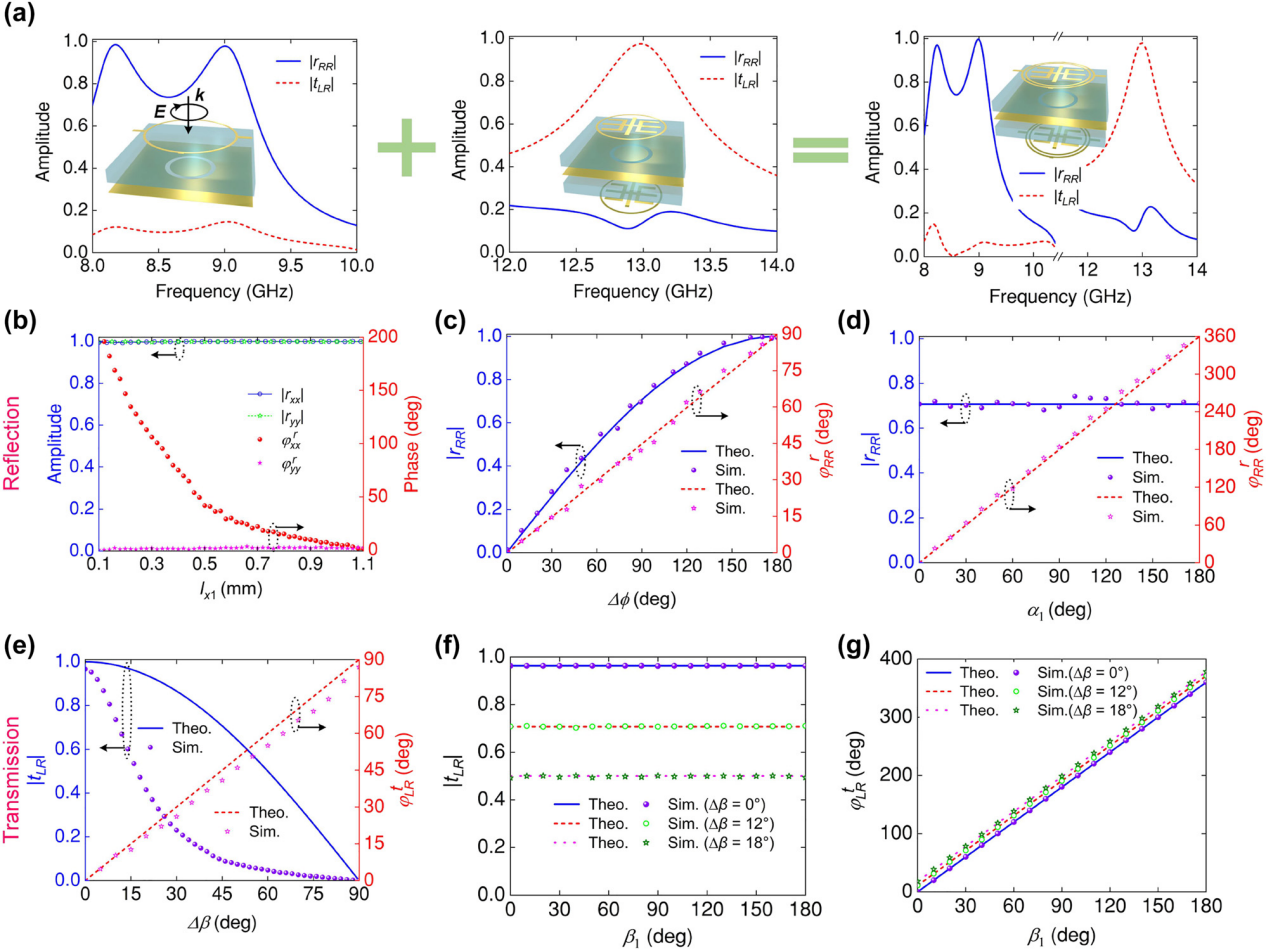
Characterization of the frequency-direction multiplexed meta-atom for full-space A–P modulation under forward RCP wave excitation. (a) The EM spectrum of different metastructures, including the external substructure, internal substructure, and entire meta-atom. (b) Reflected A–P responses of *r*_*xx*_ and *r*_*yy*_ versus the *l*_*x*1_ under orthogonal LP waves excitation at *f*_1_. Reflected A–P responses of *r*_*RR*_ as functions of parameters (c) Δ*ϕ* and (d) *α*_1_ at *f*_1_, respectively. (e) Transmissive A–P response of *t*_*LR*_ as functions of parameter Δ*β* at *f*_2_. (f)–(g) The A–P response of the *t*_*LR*_ with different Δ*β* as functions of parameter *β*_1_ at *f*_2_.

To further explain the work principle of the composite meta-atom, we divided the meta-atom into two individual substructures. Figure 3(a) plots the EM spectrum of the meta-atoms in different scenarios. For each substructure, a 180° out-of-phase condition is fulfilled for two orthogonal copolarized components at the LP wave, so that the highest efficiency can be achieved at each frequency, as shown in Figure S1(a) and (b) (Supporting Information). As is shown, the external substructure affords two R bands centered at 8.3 and 9 GHz with intensity of $\left|r_{RR}\right|$ approached one. On the contrary, the internal substructure contributes T band centered at 13 GHz with intensity of $\left|t_{LR}\right|$ above 0.97. The EM spectrum of the entire meta-atom are preserved almost the same as those that occurred in the external and internal substructure cases, revealing the crosstalk among the two modes is negligible.

Next, we investigate the A–P response of the meta-atom in R channel. Due to the mirror symmetry of the structure, the external subelement can offer an independent control of the phase responses for two orthogonal LPs. It is clearly observed from Figure 3(b) that the reflection phase $\varphi_{xx}^{r}$ changes from 200° to 0° when *l*_*x*1_ varies from 0.1 mm to 1.1 mm. At the same time, the reflection phase $\varphi_{yy}^{r}$ is nearly immune from the change of *l*_*x*1_. We then use the designed meta-atom to validate the above strategy derived from Equation (3). Fixed the parameters *l*_*y*1_ = 1.1 mm ( $\varphi_{yy}^{r}=0^{◦}$ ) and orientation angle *α*_1_ = 0° of top external subelement. Then, we only need to perform 1D parameter scan of $\varphi_{xx}^{r}$ to tailor Δ*ϕ*. The simulated A–P response versus Δ*ϕ* for *r*_*RR*_ is shown in Figure 3(c), where good agreements can be observed between simulation result and the counterpart predicted by Equation (3). It is shown that the $\left|r_{RR}\right|$ can be easily tailored from 0 to 1 by tuning Δ*ϕ*, and $\varphi_{RR}^{r}$ have a 90° phase shift. Then, we set Δ*ϕ* = 90° and rotate the element to introduce geometric phase for phase modulation. Figure 3(d) shows the simulated and theoretical calculated A–P of *r*_*RR*_, an additional reflected phase shift exists with a relationship $\varphi_{RR}^{r}=2\alpha$ while $\left|r_{RR}\right|$ is approximately 0.707. Simulation results apparently coincide with the theoretical predictions, verifying arbitrary phase manipulation of *r*_*RR*_ can be realized by *α*_1_ with negligible influence on the amplitude.

Following Equation (6), we can use our smartly designed meta-atom to implement A–P modulation in T channel. As is expected in Figure 3(e), the $\left|t_{LR}\right|$ modulation can be conducted by changing the angle difference Δ*β* = *β*_2_ − *β*_1_ at fixed *β*_1_ = 0°. The parameters *β*_1_ and *β*_2_ represent the rotation angles of the top and bottom internal subelements, respectively. The simulated $\left|t_{LR}\right|$ can be tailored in the range from 0.97 to 0, while the phase shift of $\varphi_{LR}^{t}$ changes from 0° to 88°. As a comparison, we draw the theoretical result of Equation (6) in the figure. We see the phase variation well fits the theoretical one. However, there is some deviation in the amplitude spectrum, which may be caused by coupling among different layers. Despite this, it does not affect the amplitude modulation in T channel. We quantitatively studied the influence of angle *β*_1_ on the transmission when the Δ*β* at a fixed value. In the simulation, the Δ*β* is fixed at 0°, 12°, and 18° correspond to the theoretical values of 0.97, 0.707, and 0.5, respectively. As shown in Figure 3(f) and (g), the simulated amplitude of *t*_*LR*_ keeps a consistent, and the changed phase satisfying $\varphi_{LR}^{t}=2\beta_{1}$ . The simulation results are in accordance with the theoretical results, verifying both Δ*β* and *β*_1_ can fabricate meta-atom to achieve arbitrary A–P of *t*_*LR*_. Detailed evidence about the elegant insulation among R and T modes can be inspected from Figure S1(c)–(f) (Supporting Information).

## 3 Full-space trifunctional metasurface design

Metalenses have been intensively studied due to their ability for energy harvesting and wireless power transfer. However, conventional phase-only metasurfaces typically only achieve a single focus. Multifocus responses require complicated optimization algorithms, which consumes large computing resource and time. Besides, multibeam devices capable of multichannel communication are another classic application of metasurfaces. Therefore, the generation of multibeam is important for multiple target tracking and wireless communications systems. Fortunately, the aforementioned functions can be directly realized by enabling A–P control. Here, as a proof-of-concept demonstration, we design a metasurface consisted of 26 × 26 meta-atoms to achieve three independent functionalities in full space. They are the lateral and axial bifocal metalenses in reflection modes on two sides of metasurface, and multibeam generator in transmitted mode.

Firstly, we consider the two reflection cases. The metasurface will be bifocal metalens under forward or backward RCP wave excitation at *f*_1_. The bifocal metalens can transform the incident wave into two focal spots with arbitrary position and desirable powers. The A–P distributions for designing multifocal metalens are determined by

(7) $$\begin{array}{ll} & A\left(m,n\right)e^{j\varphi \left(m,n\right)}=\overset{I}{\underset{i=1}{\sum }}A_{i}e^{jk\left(\sqrt{{\left(x\left(m,n\right)-x_{i}\right)}^{2}+{\left(y\left(m,n\right)-y_{i}\right)}^{2}+F_{i}^{2}}-F_{i}\right)} \end{array}$$

where *I* is the number of focal spots, *x*(*m*, *n*) and *y*(*m*, *n*) are the location of *mn*-th element on the *x* and *y* axes, respectively, *A*_*i*_ is the amplitude of *i*-th focal spot with focal length *F*_*i*_, *k* = 2*π*/*λ* is the wave vector in free space, *x*_*i*_ and *y*_*i*_ are positions of the *i*-th foci with respect to the lens center on the *x* and *y* axes, respectively. Take two foci as an example, we design bifocal metalens with lateral or axial aligned foci, respectively. For the lateral case, the parameters are set to (*x*_1_, *y*_1_) = (−60 mm, 0 mm), (*x*_2_, *y*_2_) = (60 mm, 0 mm), and focal length *F*_1_ = *F*_2_ = 140 mm. The amplitudes of two foci are *A*_1_:*A*_2_ = 1: $\sqrt{2}$ to predetermine the power allocation ratio as 1:2. Meanwhile, the parameters of the axial bifocal metalens are set as *F*_1_ = 60 mm, *F*_2_ = 150 mm, and *A*_1_:*A*_2_ = $\sqrt{2}$ :1.

**Figure 4:**
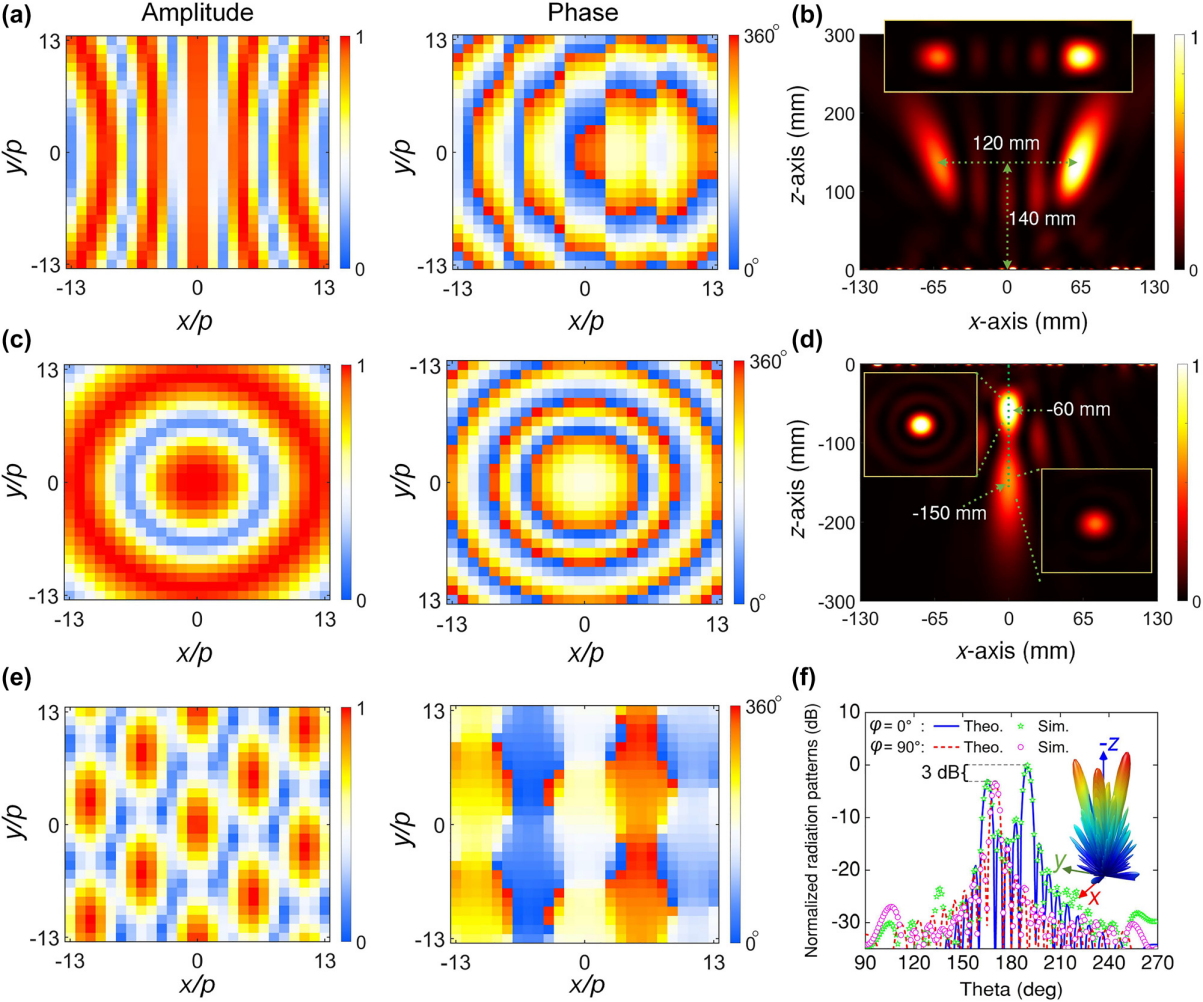
Performances of the proposed trifunctional metasurface with A–P controls in full space. The required A–P distributions for (a) lateral and (c) axial bifocal metalenses, and (e) multibeam generator. The simulated normalized power distributions of RCP *E*-field on the *xoz* cutting plane under (b) forward and (d) backward RCP wave excitation at *f*_1_. (f) The theoretical and simulated 2D normalized scattering pattern in the *φ* = 0° (*xoz*) and *φ* = 90° (*yoz*) planes under forward RCP wave excitation at *f*_2_. The inset shows the simulated 3D far-field radiation pattern.

The spatial distributions of the required A–P imparted by lateral and axial bifocal metalenses are illustrated in Figure 4(a) and (c), respectively. These A–P distributions can be achieved by the design of the length *l*_*xi*_ and rotation angle *α*_*i*_ of the top and bottom external subelements with fixed *l*_*yi*_ = 1.1 mm (*i* = 1, 2). Figure 4(b) and (d) show the simulated RCP *E*-field power profiles when the metasurface is excited by forward and backward RCP wave at *f*_1_, respectively. Two focal spots with different offset distances and different focal lengths can be observed in the upper and lower half spaces of the *xoz* plane. We see the field intensity of right spot in lateral bifocal metalens is significantly stronger than that of the left point, and upper spot in axial bifocal metalens stronger than lower spot. The simulated results are consistent with the theoretical designs. The insets of the Figure 4(b) and (d) display magnified details at the focal plane *z* = 140, −60, and −150 mm for the lateral and axial bi-foci cases, respectively. The energy decays quickly and is of extremely weak intensity in other spaces, facilitating a high efficiency. In addition, the focusing efficiency (defined as the ratio of the power focused to the desired spots to the input power) is calculated at ≈ 64 %, 41 %, and 34 % for the three focal planes, respectively [57]. Therein, the energy of the focusing spot can be obtained by the sum of simulated intensity in the area of focal spot. The incident energy is obtained by the same process in the focal plane without placing the metasurface [58], [59]. Besides, acceptable polarization purities are observed by comparing the simulated co- and cross-polarization components of the two metalenses, see more details in Figure S2 (Supporting Information).

Finally, as for the transmission case, the metasurface will be multibeam generator under forward RCP wave excitation at *f*_2_. The A–P modulation is necessary to generate multibeam with arbitrary pointing directions and relative power allocations. The required A–P patterns on the multibeam generator aperture can be expressed as

(8) $$A\left(m,n\right)e^{j\varphi \left(m,n\right)}=\overset{L}{\underset{l=1}{\sum }}A_{l}e^{-jpk\sin\left(\theta_{l}\right)\left[\cos\left(\varphi_{l}\right)m+\sin\left(\varphi_{l}\right)n\right]}$$

where *L* is the number of beams, *θ*_*l*_, *φ*_*l*_, and *A*_*l*_ are the elevation angle, azimuth angle, and the amplitude of the *l*-th beam, respectively, and *p* is the periodicity of each element. Without loss of generality, a case of three spatial radiation beams with different powers is selected as an example. The radiation directions of the three beams are (*θ*, *φ*) = (165°, 0°), (190°, 0°), and (170°, 90°). Simultaneously, we set the parameters *A*_1_:*A*_2_:*A*_3_ = 1: $\sqrt{2}$ :1 to predetermine the radiation power ratio as 1:2:1.

Figure 4(e) shows the theoretically calculated A–P distribution for the three-beam generator, which can be easily obtained by the angles *β*_1_ and Δ*β* of the internal subelement. Under the normal incidence of plane wave, the scattering pattern from the metasurface with *M* × *N* elements can be calculated as:

(9) $$\begin{array}{ll} & E_{\text{total}}\left(\theta ,\varphi \right)\\ & \;=\overset{M}{\underset{m=1}{\sum }}\overset{N}{\underset{n=1}{\sum }}\left(A^{mn}e^{-j\varphi^{mn}}\cdot e^{-jpk\sin\theta \left[\left(m-1/2\right)\cos\varphi +\left(n-1/2\right)\sin\varphi \right]}\right) \end{array}$$

where *θ* and *φ* are the elevation and azimuth angles of an arbitrary direction, *A* ^*mn*^ and *φ* ^*mn*^ are the amplitude and phase responses of the *mn*-th element. The simulated 3D far-filed scattering pattern at *f*_2_ is presented in the inset of Figure 4(f). Three scattering beams can be obtained with different spatial directions and powers. Figure 4(f) plots the theoretical and simulated 2D normalized scattering patterns in *φ* = 0° (*xoz*) and *φ* = 90° (*yoz*) planes. We observe that three anomalous direction angles appear at around (*θ*, *φ*) = (165°, 0°), (190°, 0°), and (170°, 90°). In addition, the scattering power of the beam with deflection angle *θ* = 190° is 3 dB higher than that of the other two beams along the *z*-axis. The simulated results are good in agreement with the theoretical prediction, demonstrating the metasurface can generate multibeams with arbitrary directions and power allocations. Besides, we may even obtain more sophisticated radiation patterns, such as flat-top beam, cosecant squared beam, and so on.

## 4 Experimental verification

**Figure 5:**
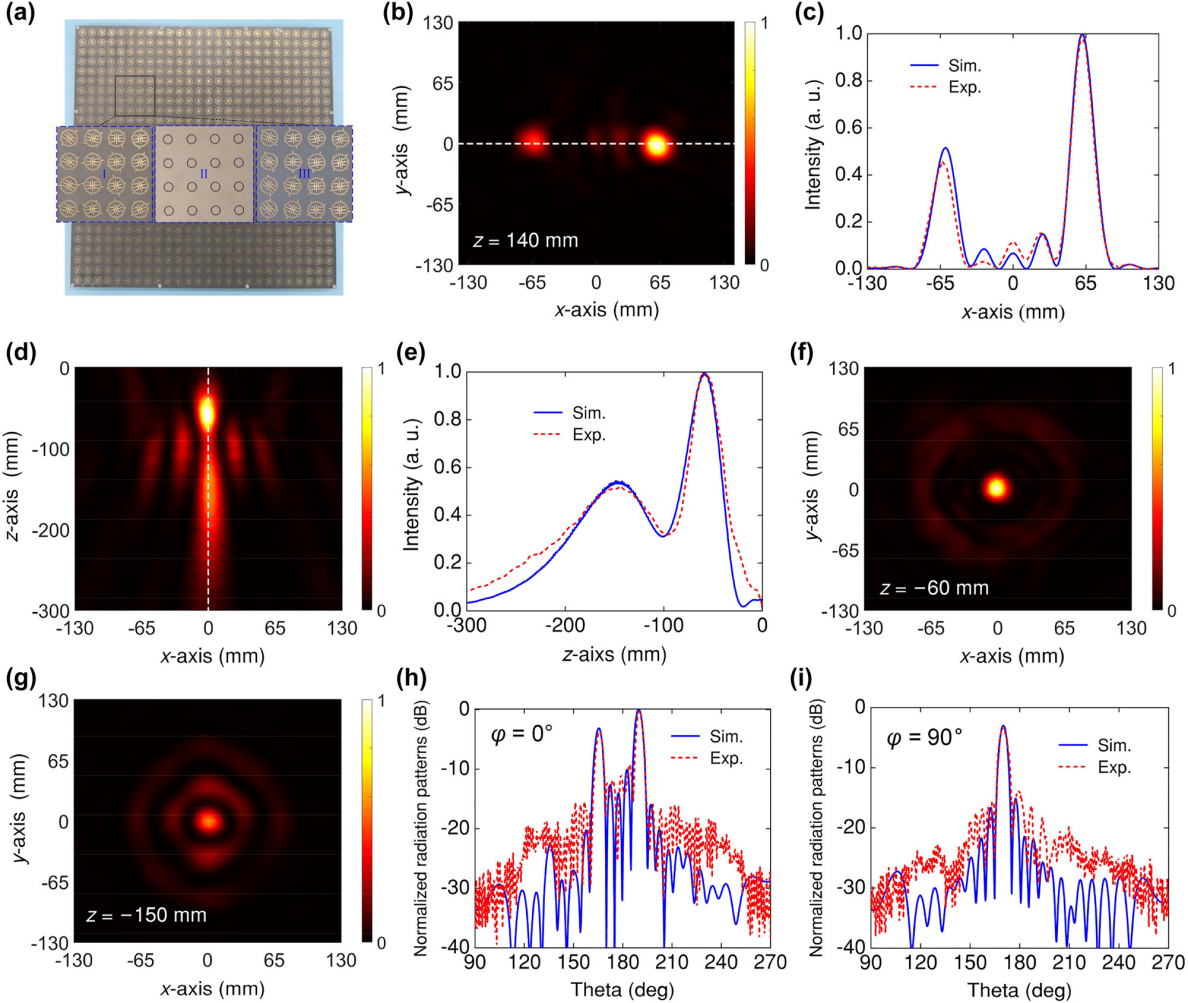
Experimental characterization of the fabricated trifunctional metasurface. (a) The photograph of the fabricated sample, and the insets show the enlarged view of different layers. The measured normalized power distribution of RCP *E*-field power of (b) lateral bifocal metalens in *xoy* plane and axial bifocal metalens in (d) *xoz* plane, (f) *z* = −60 mm, and (g) *z* = −150 mm planes at 9 GHz. The simulated and measured power scanned along the line (c) *y* = 0 mm of lateral bifocal metalens in *xoy* plane, and (e) *x* = 0 mm of axial bifocal metalens in *xoz* plane. The measured and simulated 2D normalized scattering pattern for the RCP incidence in the (h) *φ* = 0° and (i) *φ* = 90° planes at 13 GHz.

To experimentally validate the above-designed trifunctional metasurface, we fabricated a sample using standard printed circuit board (PCB) technology. The sample metasurface consisted of 26 × 26 meta-atoms, as shown in Figure 5(a). Two layers of F4B substrates are fabricated with the same dimension and fixed together by several plastic screws. The insets show some detailed information about different layers of the selected area on the sample. More details about the structures in different layers and measurement setup are shown in Figures S3 and 4 of Supporting Information, respectively.

We first measured the bifocal performance of the lateral bifocal metalens. The measured RCP *E*-field power profile on *xoy* plane at 9 GHz is shown in Figure 5(b). The field is normalized to their global maximum. Two focal spots with different intensities can be clearly observed at the preset focal plane. For a clearer comparison, the simulated and measured normalized RCP *E*-field power scanned along the line of *y* = 0 mm in the focal plane is plotted in Figure 5(c). As expected, two focal spots approximately located at (−60 mm, 0 mm, 140 mm) and (60 mm, 0 mm, 140 mm) with the intensity ratio about 1:2. The measured focusing efficiency is 60 % and the two focal spot sizes characterized by half power beamwidth are about 0.63*λ* (*λ* = 33.3 mm).

On the other side of the metasurface, the measured RCP *E*-field power profile of the axial bifocal metalens is shown in Figure 5(d). As expected, two focal spots distributed vertically. The intensity distribution along the optical axis is shown in Figure 5(e). We see two peaks in EM power around *z* = −60 mm and *z* = −150 mm with the ratio about 2:1. It can be seen that the experimental result is in good agreement with the simulation and confirming the design goals. To clearly illustrate the focusing effect, Figure 5(f) and (g) show the measured RCP *E*-field power profiles on the focal plane *z* = −60 and *z* = −150 mm, respectively. It shows that the plane wave has been focused through the metasurface, and the two focal-spot sizes are 0.54*λ* and 0.6*λ*. The measured focusing efficiency in the two focal planes are 35 % and 27 %, respectively.

Finally, the performance of multibeams was measured in microwave anechoic. Figure 5(h) and (i) show the measured 2D normalized scattering patterns in *φ* = 0° and *φ* = 90° planes at 13 GHz, respectively. The measured powers are normalized to their maximums. It is clear that three oblique beams transmitted from the metasurface appears at around (*θ*, *φ*) = (165°, 0°), (190°, 0°), and (170°, 90°). In addition, the measured power of the beam with deflection angle *θ* = 190° is 3 dB higher than that of the other two beams, indicating that the power ratio of the three beams about 1:2:1. The measured and simulated results are consistent, indicating that the incident plane wave is split into three beams with predetermined directions and power distributions. The discrepancies between the measurement and simulation may be due to the fabrication and measurement tolerances. The results displayed in Figure 5 prove the metasurface has the ability to arbitrarily modulate near-field and far-field power in full space.

To illustrate the advantages of our metasurface, Table 1 compares the key characteristics of the proposed work with that of previous work. Such a comparison result indicates that ours has the advantages of the simple structure, smallest footprint, low profile, and high efficiency. Furthermore, our strategy can control the A–P of EM waves in three independent channels. The advantages of our metasurface enable us to design some sophisticated functions with large capacity.

**Table 1:** Comparison of different full-space metasurfaces with A–P control.^a^

Refs	Functions	Metal layers	Meta-atom size (*λ*_0_)	Efficiencies	Polarization	Methods	Frequency (GHz)
[48]	Two	Three	0.57 × 0.57 × 0.34	R: 0–0.9 T: 0–0.55	LP	Polarization conversion	7&17
[54]	Two	Six	0.63 × 0.63 × 0.45	R: 0–1 T: 0–1	CP	Hybrid phase	8.7&15.8
[55]	Two	Two	0.43 × 0.43 × 0.15	Discrete	CP	Removing resonator	10&15
[56]	Three	Four	0.57 × 0.57 × 0.71	R: 0–1 T1: 0–0.9 T2: 0–0.85	CP	Dual geometric phase	7&10.2&15.7
This work	Three	Three	0.43 × 0.43 × 0.087	R1: 0–1 R2: 0–1 T: 0–1	CP	Hybrid phase & dual geometric phase	9&13

^a^Note: The meta-atom size is evaluated relative to the wavelength at the high frequency.

## 5 Conclusions

In summary, we have proposed a full-space trifunctional metasurface with individual and simultaneous A–P control for CP wave. In this regard, a novel type of meta-atom composed of three metal layers and two substrates was devised. The A–P responses in T and R modes can be independently tuned with negligible crosstalk due to the middle bandpass frequency selective surface structure. The hybrid phase and dual geometric phases strategies are applied to achieve independent A–P control of CP wave in R and T channels, respectively. As proof of concept, a metadevice was devised to integrate three advanced functions in full space, showcasing lateral and axial bifocal metalenses with desired intensity distribution at *f*_1_, and multibeam generator with desired spatial scatterings and power allocations at *f*_2_. Our trifunctional metasurface was experimentally verified at microwave frequencies, and the results are in good agreement with the design. We firmly believe that our proposed methodology for designing the large-capacity metadevices will have bright application prospects in beams shaping, high-quality holography, and multiple target tracking using full-space A–P control.

## Supplementary Material

Supplementary Material Details
