# Supplementary material for: Full-space trifunctional metasurface with independent control of amplitude and phase for circularly polarized waves
Source: Nanophotonics. 2024 Oct 23;13(24):4471–81. doi: 10.1515/nanoph-2024-0441 (PMC11636479; doi:10.1515/nanoph-2024-0441)
Supplement: Supplementary file 1 — Supplementary Material Details [file j_nanoph-2024-0441_suppl_001.pdf]

Supporting Information

Full-space Trifunctional Metasurface with Independent Control of Amplitude and Phase for Circularly Polarized Waves

Xi Ming Li[1,2,3,†], *Yuan Zhao*[2,†], *Ren Pan Lu*[2], *Xiao Feng Sun*[2], *Zhao Yang*[2], *Hai Dan He*[3], *Yan Hui Liu*[1,*], *and Guo Hong Du*[2,*]

[1] School of Electronic Science and Engineering, University of Electronic Science and Technology of China, Chengdu 611731, China

[2] College of Electronic Engineering, Chengdu University of Information Technology, Chengdu 610225, China

[3] Southwest China Institute of Electronic Technology, Chengdu 610036, China

*E-mail: yhliu@uestc.edu.cn

*E-mail: dghong@cuit.edu.cn

[†]These authors contributed equally to this work.

The Supporting Information includes:

- 1. Theory for CP reflection and transmission coefficients
- 2. Hybrid phase approach for A-P modulation in reflection channel
- 3. Dual geometric phases approach for A-P modulation in transmission channel
- 4. Additional results for the meta-atom
- 5. Polarization purities of lateral and axial bifocal metalenses
- 6. Detailed layout for the trifunctional metasurface
- 7. Experimental Methods

1. Theory for CP reflection and transmission coefficients

For a meta-atom with mirror symmetry, the relationship between the incident and reflected (transmissive) electric field can be expressed as

$$\begin{bmatrix} E_x^r \\ E_y^r \end{bmatrix} = \mathbf{R} \begin{bmatrix} E_x^i \\ E_y^i \end{bmatrix} = \begin{bmatrix} r_{xx} & 0 \\ 0 & r_{yy} \end{bmatrix} \begin{bmatrix} E_x^i \\ E_y^i \end{bmatrix}, \tag{S1a}$$

$$\begin{bmatrix} E_x^t \\ E_y^t \end{bmatrix} = \mathbf{T} \begin{bmatrix} E_x^i \\ E_y^i \end{bmatrix} = \begin{bmatrix} t_{xx} & 0 \\ 0 & t_{yy} \end{bmatrix} \begin{bmatrix} E_x^i \\ E_y^i \end{bmatrix}, \tag{S1b}$$

where **R** and **T** is the reflection and transmission matrix, respectively, r_{xx}, r_{yy}, t_{xx}, and t_{yy} are the reflection and transmission coefficients with polarizations along two principal axes x and y.

If the meta-atom is rotated by an angle α along the z-axis, the linear reflection complex Jones $\mathbf{R}(\alpha)$ and transmission complex Jones matrix $\mathbf{T}(\alpha)$ can be formulated as

$$\mathbf{R}(\alpha) = \mathbf{M}^{-1}(\alpha)\mathbf{R}\mathbf{M}(\alpha), \tag{S2a}$$

$$\mathbf{T}(\alpha) = \mathbf{M}^{-1}(\alpha)\mathbf{T}\mathbf{M}(\alpha), \tag{S2b}$$

where $\mathbf{M}(\alpha) = \begin{bmatrix} \cos(\alpha) & \sin(\alpha) \\ -\sin(\alpha) & \cos(\alpha) \end{bmatrix}$ describes the rotation matrix. The incident, reflected and transmissive CP components can be expressed with the linear polarization components by

$$\begin{bmatrix} E_L^i \\ E_R^i \end{bmatrix} = \mathbf{C} \begin{bmatrix} E_x^i \\ E_y^i \end{bmatrix} = \frac{1}{\sqrt{2}} \begin{bmatrix} 1 & j \\ 1 & -j \end{bmatrix} \begin{bmatrix} E_x^i \\ E_y^i \end{bmatrix}, \tag{S3a}$$

$$\begin{bmatrix} E_L^r \\ E_R^r \end{bmatrix} = \mathbf{C}' \begin{bmatrix} E_x^r \\ E_y^r \end{bmatrix} = \frac{1}{\sqrt{2}} \begin{bmatrix} 1 & -j \\ 1 & j \end{bmatrix} \begin{bmatrix} E_x^r \\ E_y^r \end{bmatrix}, \tag{S3b}$$

$$\begin{bmatrix} E_L^t \\ E_R^t \end{bmatrix} = \mathbf{C} \begin{bmatrix} E_x^t \\ E_y^t \end{bmatrix} = \frac{1}{\sqrt{2}} \begin{bmatrix} 1 & j \\ 1 & -j \end{bmatrix} \begin{bmatrix} E_x^t \\ E_y^t \end{bmatrix}, \tag{S3c}$$

in which E_L^χ and E_R^χ ($\chi = i, r, t$) represent the LCP and RCP components of the incident, reflected and transmissive fields, respectively. Due to the opposite directions of propagation between the incident and reflected waves, the rotation directions of the CP waves are also opposite. Then combining the above equations, there is

$$\begin{bmatrix} E_L^r \\ E_R^r \end{bmatrix} = \mathbf{C'M}^{-1}(\alpha)\mathbf{RM}(\alpha)\mathbf{C}^{-1}\begin{bmatrix} E_L^i \\ E_R^i \end{bmatrix}$$

$$= \frac{1}{2}\begin{bmatrix} (r_{xx}-r_{yy})\cdot e^{j\cdot 2\alpha} & r_{xx}+r_{yy} \\ r_{xx}+r_{yy} & (r_{xx}-r_{yy})\cdot e^{-j\cdot 2\alpha} \end{bmatrix}\begin{bmatrix} E_L^i \\ E_R^i \end{bmatrix}, \quad \text{(S4a)}$$

$$\begin{bmatrix} E_L^t \\ E_R^t \end{bmatrix} = \mathbf{CM}^{-1}(\alpha)\mathbf{RM}(\alpha)\mathbf{C}^{-1}\begin{bmatrix} E_L^i \\ E_R^i \end{bmatrix}$$

$$= \frac{1}{2}\begin{bmatrix} t_{xx}+t_{yy} & (t_{xx}-t_{yy})\cdot e^{j\cdot 2\alpha} \\ (t_{xx}-t_{yy})\cdot e^{-j\cdot 2\alpha} & t_{xx}+t_{yy} \end{bmatrix}\begin{bmatrix} E_L^i \\ E_R^i \end{bmatrix}, \quad \text{(S4b)}$$

2. Hybrid phase approach for A-P modulation in reflection channel

The co-polarized reflection coefficients of reflective meta-atom are all enhanced to 100% for a lossless case ($|r_{xx}|=|r_{yy}|=1$), if the element is mirror symmetric with respect to the x and y axes. Thus, the A–P response of each CP channel from Equation (S4a) is presented as:

$$r_{LR} = r_{RL} = \frac{1}{2}(e^{j\varphi_{xx}} + e^{j\varphi_{yy}}) = \cos\frac{\varphi_{xx}-\varphi_{yy}}{2}\cdot e^{j\frac{(\varphi_{xx}+\varphi_{yy})}{2}}, \quad \text{(S5a)}$$

$$r_{RR} = \frac{1}{2}(e^{j\varphi_{xx}} - e^{j\varphi_{yy}})\cdot e^{i2\alpha} = \sin\frac{\varphi_{xx}-\varphi_{yy}}{2}\cdot e^{j[(\varphi_{xx}+\varphi_{yy}+\pi)/2+2\alpha]}, \quad \text{(S5b)}$$

$$r_{LL} = \frac{1}{2}(e^{j\varphi_{xx}} - e^{j\varphi_{yy}})\cdot e^{-i2\alpha} = \sin\frac{\varphi_{xx}-\varphi_{yy}}{2}\cdot e^{j[(\varphi_{xx}+\varphi_{yy}+\pi)/2-2\alpha]}, \quad \text{(S5c)}$$

If incident wave is assumed as RCP wave, A–P response of co-polarized reflection coefficient can be derived from Equation (S5b) and written as:

$$\left|r_{RR}(\alpha)\right| = \left|\sin(\frac{\Delta\phi}{2})\right|, \quad \text{(6a)}$$

$$\varphi_{RR}^r(\alpha) = \frac{1}{2}(\Delta\phi+\pi) + \varphi_{yy}^r + 2\alpha, \quad \text{(6b)}$$

in which $\Delta\phi = \varphi_{xx}^r - \varphi_{yy}^r$.

3. Dual geometric phases approach for A-P modulation in transmission channel

Supposing a transmissive meta-atom composed of mirror symmetric dual-metal layers, and the rotation angles of the two metal pattern layers are β_1 and β_2, respectively. According to transfer-

matrix approach, the total transmissive matrix of the composite meta-atom can be derived as

$$T_{cir}^{total} = T_{cir}(\beta_1) \cdot T_d \cdot T_{cir}(\beta_2), \tag{7}$$

$$T_{cir}(\beta) = \frac{1}{2}\begin{bmatrix} t_{xx} + t_{yy} & (t_{xx} - t_{yy}) \cdot e^{j \cdot 2\beta} \\ (t_{xx} - t_{yy}) \cdot e^{-j \cdot 2\beta} & t_{xx} + t_{yy} \end{bmatrix}, \tag{8}$$

where $T_{cir}(\beta)$ and $T_d = [1,0;0,1]$ are the transmission coefficient of a single metal layer and isotropic dielectric substrate in CP basis, respectively. Then, the total cross-polarized transmissive coefficients can be derived as

$$T_{LR}^{total} = \frac{1}{4}\left[(t_{xx})^2 - (t_{yy})^2\right] t_d (e^{j2\beta_1} + e^{j2\beta_2}), \tag{9a}$$

$$T_{RL}^{total} = \frac{1}{4}\left[(t_{xx})^2 - (t_{yy})^2\right] t_d (e^{-j2\beta_1} + e^{-j2\beta_2}), \tag{9b}$$

Since $(t_{xx})^2 t_d = t_{xx}^{total}$ and $(t_{yy})^2 t_d = t_{yy}^{total}$. Simultaneously, the anisotropic conditions of $|t_{xx}^{total}| = |t_{yy}^{total}| = 1$ and $\varphi_{xx}^{total} - \varphi_{yy}^{total} = \pm\pi$ are required to engineer maximum cross-CP conversion efficiency, Equation (9) can be further simplified as

$$T_{LR}^{total} = \frac{1}{2}(e^{j2\beta_1} + e^{j2\beta_2}) = \cos(\Delta\beta) e^{j(2\beta_1 + \Delta\beta)}, \tag{10a}$$

$$T_{RL}^{total} = \frac{1}{2}(e^{-j2\beta_1} + e^{-j2\beta_2}) = \cos(\Delta\beta) e^{-j(2\beta_1 + \Delta\beta)}, \tag{10b}$$

where the angle difference $\Delta\beta = \beta_2 - \beta_1$.

4. Additional results for the meta-atom

To further explain the high efficiency of the meta-atom in both R and T modes, we studied the scattering characteristics of the two individual substructures. Figure S1(a) and (b) illustrate the scattering coefficients of the external and internal substructures, respectively, for two orthogonal linear polarized waves at normal incidence. The figures show that the anisotropic conditions of $|r_{xx}| = |r_{yy}| = 1$ and $\varphi_{xx}^r - \varphi_{yy}^r = \pm\pi$ in R case while $|t_{xx}| = |t_{yy}| = 1$ and $\varphi_{xx}^t - \varphi_{yy}^t = \pm\pi$ in T case are satisfied at 9 and 13 GHz, respectively. The results guarantee high efficiency of the composite meta-atom in R and T channels, when it under CP wave illumination.

Crosstalk among different sub-elements can certainly affect the performance of an entire meta-

atom. As an illustration, we explore the mutual influence of A–P responses in R and T channels. For example, we set $\Delta\beta = 12°$ and $\beta_1 = 0°$ ($t_{LR} = 0.707$), the simulated A–P responses of the meta-atom versus the $\Delta\phi$ and α_1 at 13 GHz are shown in Figure S1(c) and (d). We see that the simulated A–P profiles of t_{LR} remain stable when $\Delta\phi$ and α_1 are changed. The theoretical A-P values in the figures are set as the same as the simulated A-P of the meta-atom with $\Delta\beta = 12°$ and $\beta_1 = 0°$, which are not affected by $\Delta\phi$ and α_1 based on the Equation (6). Meanwhile, we set $\Delta\phi = 90°$ and $\alpha_1 = 0°$ ($r_{RR} = 0.707$), the simulated A–P responses of r_{RR} hardly change as varying the $\Delta\beta$ and β_1 at 9 GHz (Figure S1(e) and (f)). According to Equation (6), the A-P responses of r_{RR} are not affected by $\Delta\beta$ and β_1. The results show good isolation of the composite meta-atom between R and T modes.

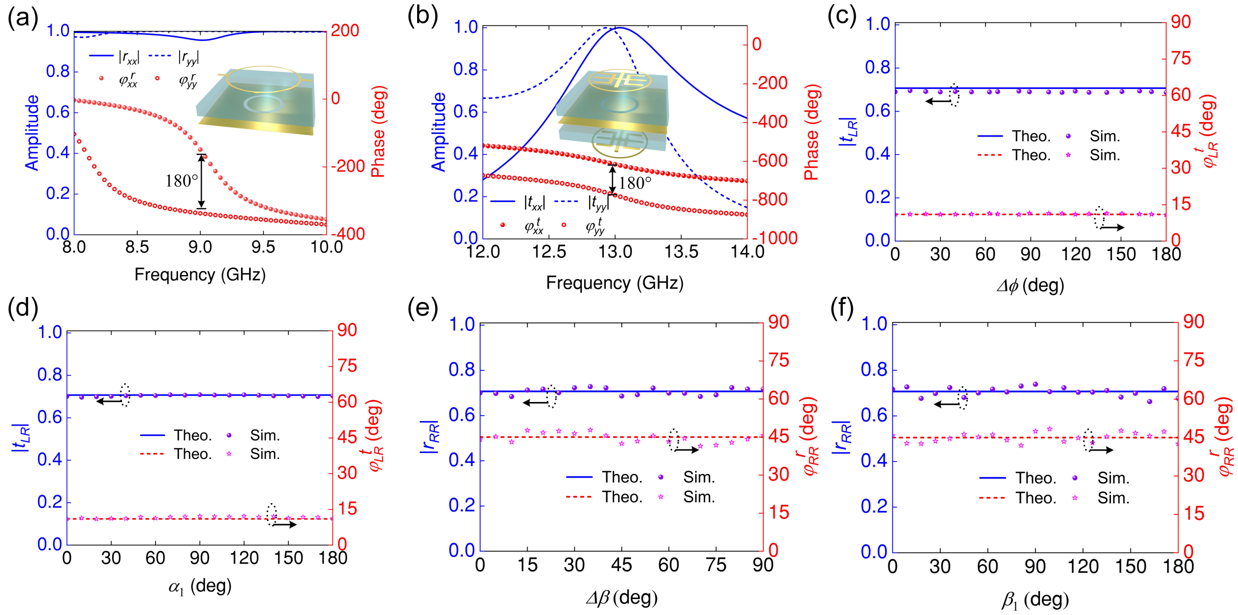

Figure S1. EM response of the (a) external and (b) internal substructures under excitation of orthogonal LP waves. The transmission coefficients vs (c) $\Delta\phi$ and (d) α_1 with $\Delta\beta = 12°$ at 13 GHz. The reflection coefficients vs (e) $\Delta\beta$ and (f) β_1 with $\Delta\phi = 90°$ at 9 GHz.

5. Polarization purities of lateral and axial bifocal metalenses

To evaluate the polarization purity of the lateral bifocal metalens, Figure S2(a) and (b) show the simulated power distributions of RCP and LCP *E*-field, respectively, when the lateral bifocal metalens is excited by forward RCP wave at 9 GHz. The simulated powers are normalized to their maximums. It can be clearly seen that the co-polarization power is stronger than the cross-

polarization on the *xoz* cutting plane. The ratio of the maxima of the co-polarization component to the cross-polarization component is about 17, indicating that the lateral bifocal metalens has acceptable polarization purity. Similarly, the simulated power distributions of RCP and LCP *E*-field of the axial bifocal metalens on the *xoz* cutting plane are shown in Figure S2(c) and (d), respectively. The RCP component of the reflected field is almost focused on two focal spots, however, the LCP component is very weak. The ratio of the maximum value of the RCP component to the LCP component is about 15. Therefore, the designed axial bifocal metalens has acceptable polarization purity.

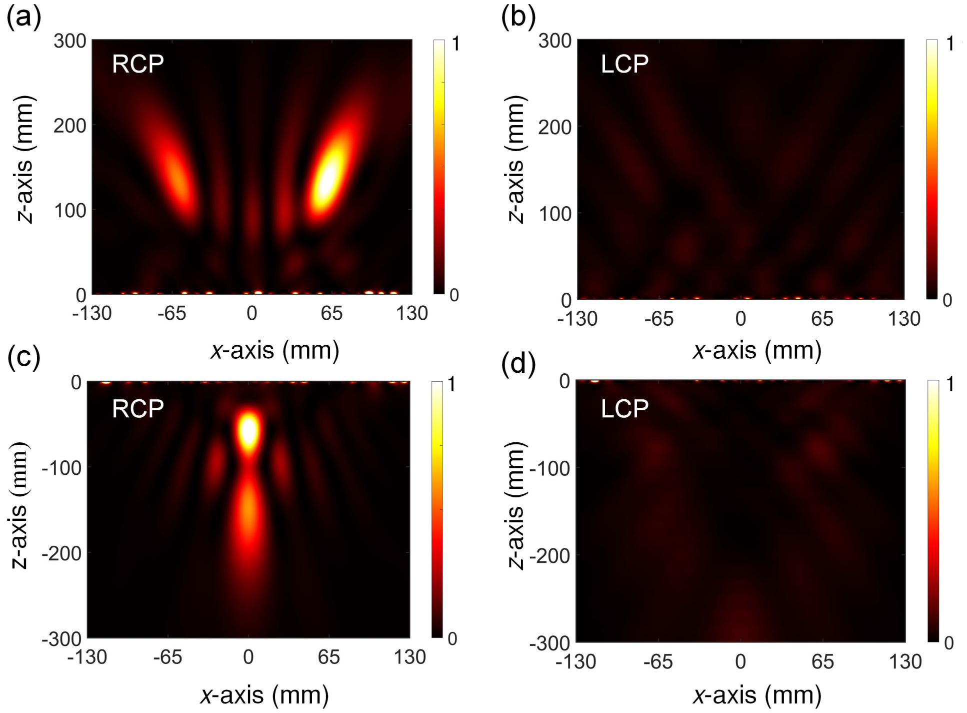

Figure S2. The simulated normalized power distributions of (a) RCP and (b) LCP *E*-field of the lateral bifocal metalens, and (c) RCP and (d) LCP *E*-field of the axial bifocal metalens on the *xoz* cutting plane at 9 GHz.

6. Detailed layout for the trifunctional metasurface

Figure S3(a)-(c) show the arrangement of the meta-atom parameters (l_{x1}, α_1, l_{x2}, α_2, β_1, and β_2), which corresponds to the required A–P distribution in Figure 4(a)-(c), respectively. Among them, Figure S3(a) and (b) show the parameter patterns (the length l_{xi} and rotation angle α_i, i = 1, 2) of the top layer external sub-elements, respectively. Figure S3(c) shows the rotation angle patterns

(β_1 and β_2) of the top and bottom layer internal sub-elements. Corresponding layouts of the trifunctional metasurface and their enlarged view are shown in Figure S3(d)-(f).

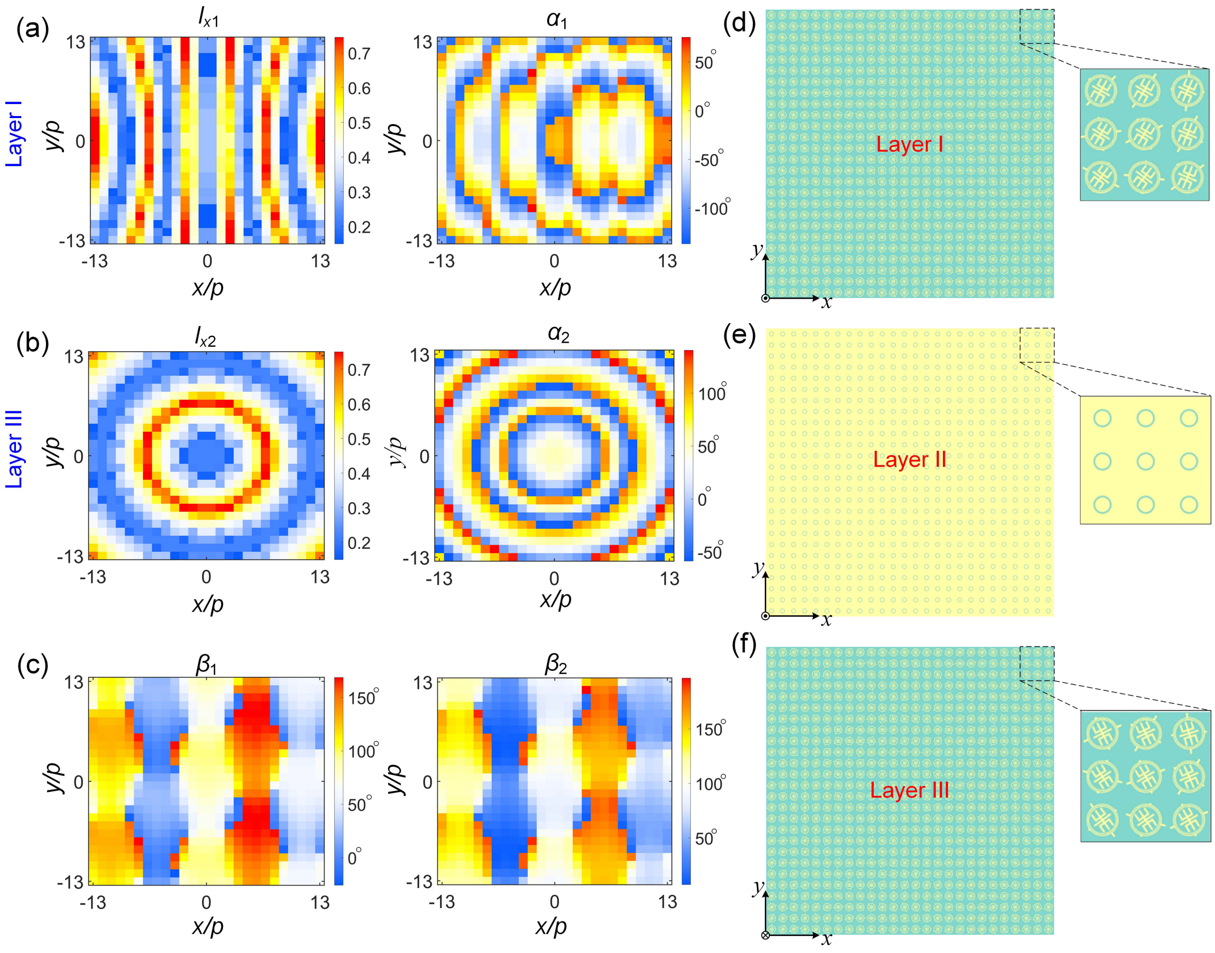

Figure S3. The distributions of the length l_{xi} and rotation angle α_i (i = 1, 2) of the metasurface on the (a) top and (b) bottom layer external sub-elements. (c) The distributions of rotation angle (β_1 or β_2) of the top and bottom layer internal sub-elements. (d)-(f) The full layout of the top, middle and bottom layer of the trifunctional metasurface.

7. Experimental Methods

In near-field experiments, the 3D near-field scanning system is used to measure the energy intensity, as shown in Figure S4(a). The fabricated sample was launched by an RCP horn with a voltage-standing-wave ratio less than 1.8 and an axial ratio less than 3.3 across 8–18 GHz. The distance between the metasurface and the transmitting antenna was about 1.5 m to guarantee a

quasi-plane plane wavefront. Then intensity distribution of the reflected wave is captured by a small-aperture spiral antenna with an axial ratio smaller than 1.8 dB from 6 to 18 GHz, which functioning as the RCP probe and connected to a vector network analyzer. In far-field experiments, the scattering characterization was carried out using an automatically moved 2D rotary table, as shown in Figure S4(b). The sample and an RCP source antenna were mounted on a rotary table, and the distance between the measured sample to the transmitter is 1.5 m. The sample is surrounded by aluminum foil to reduce the impact of the incidence on the transmitted wave. An LCP antenna was utilized as the receiver and connected to a vector network analyzer to detect the transmitted intensity.

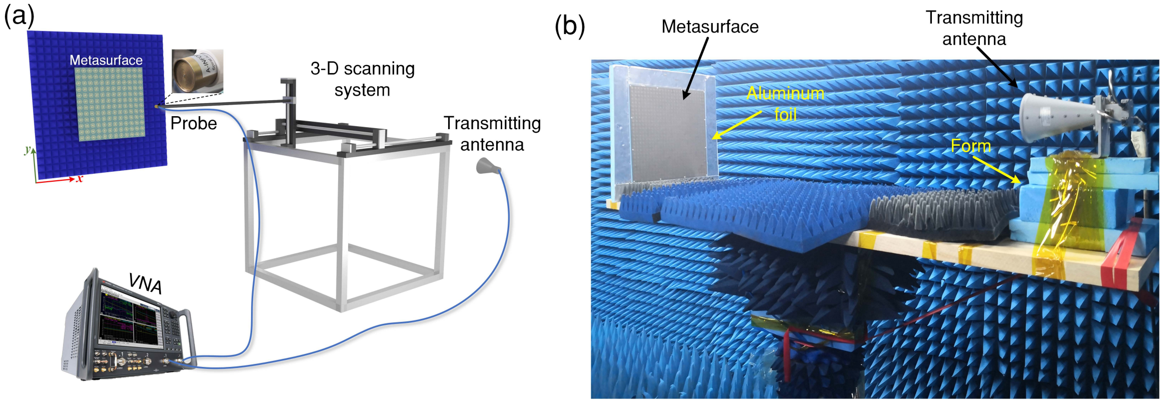

Figure S4. The experimental setup for (a) near-field and (b) far-field measurements.
